# Reducing stunting by improving maternal, infant and young child nutrition in regions such as South Asia: evidence, challenges and opportunities

**DOI:** 10.1111/mcn.12282

**Published:** 2016-05-17

**Authors:** Kathryn G. Dewey

**Affiliations:** ^1^ Department of Nutrition and Program in International and Community Nutrition University of California, Davis Davis California USA

**Keywords:** child growth, complementary feeding, maternal nutrition, micronutrient malnutrition, nutritional interventions, stunting

## Abstract

Meeting the high nutrient needs of pregnant and lactating women and their young children in regions such as South Asia is challenging because diets are dominated by staple foods with low nutrient density and poor mineral bioavailability. Gaps in nutritional adequacy in such populations probably date back to the agricultural revolution ~10 000 years ago. Options for improving diets during the first 1000 days include dietary diversification and increased intake of nutrient‐rich foods, improved complementary feeding practices, micronutrient supplements and fortified foods or products specifically designed for these target groups. Evidence from intervention trials indicates that several of these strategies, both prenatal and post‐natal, can have a positive impact on child growth, but results are mixed and a growth response is not always observed. Nutrition interventions, by themselves, may not result in the desired impact if the target population suffers from frequent infection, both clinical and subclinical. Further research is needed to understand the mechanisms underlying both prenatal and post‐natal growth restriction. In the meantime, implementation and rigorous evaluation of integrated interventions that address the multiple causes of stunting is a high priority. These intervention packages should ideally include improved nutrition during both pregnancy and the post‐natal period, prevention and control of prenatal and post‐natal infection and subclinical conditions that restrict growth, care for women and children and stimulation of early child development. In regions such as South Asia, such strategies hold great promise for reducing stunting and enhancing human capital formation.

**Key messages**

- Meeting the high nutrient needs of pregnant and lactating women and their young children in regions such as South Asia is challenging because diets are dominated by staple foods with low nutrient density and poor mineral bioavailability.
- Gaps in nutritional adequacy in such populations probably date back to the agricultural revolution ~10 000 years ago.
- Options for improving diets during the first 1000 days include dietary diversification and increased intake of nutrient‐rich foods, improved complementary feeding practices, micronutrient supplements and fortified foods or products specifically designed for these target groups.
- Evidence from intervention trials indicates that several of these strategies, both prenatal and post‐natal, can have a positive impact on child growth. However, there is considerable heterogeneity in growth response to such interventions.
- Nutrition interventions should cover both pregnancy and the post‐natal period and are likely to have a greater impact on child growth if they are delivered as part of a package that addresses the multiple causes of stunting, including prevention and control of prenatal and post‐natal infection and subclinical conditions that restrict growth, care for women and children and stimulation of early child development.

## Introduction

It is widely recognized that the key ‘window of opportunity’ for reducing stunting is the ~1000 days from conception until 2 years of age (Victora *et al*. 2010, http://www.thousanddays.org). Assuring adequate maternal nutrition prior to conception is also likely to be important (Bhutta *et al*. 2013a; Prentice *et al*. 2013). This paper will briefly discuss the challenges to meeting nutrient needs during the first 1000 days, the various strategies that can be used to improve nutrient intake in these target groups, the evidence for an impact on linear growth or stunting from prenatal and post‐natal nutrition interventions and the need for integrated approaches that address the multifactorial aetiology of stunting, with an emphasis on South Asian populations.

## Challenges to meeting nutrient needs during the first 1000 days

Nutrient requirements are high during pregnancy and lactation because of the need to support fetal growth and production of breast milk. In comparison with a non‐pregnant, non‐lactating woman, energy needs are 13% higher during pregnancy and 25% higher during lactation, and protein needs are 54% higher during both periods (Institute of Medicine 2006). For several micronutrients, the relative increase in recommended intake is ≥50%, such as for folate and iron during pregnancy and for vitamin A, vitamin C, vitamin B6, iodine and zinc during lactation. This poses a challenge because women in low‐income settings often have limited access to nutrient‐dense foods.

Children under 2 years of age also have high nutrient needs to support growth and development. Moreover, breastfed infants typically consume relatively small amounts of foods other than breast milk (Dewey & Brown 2003). Infants need complementary foods with much higher nutrient density (amount of each nutrient per 100 kcal) than is required for adult diets. For example, per 100 kcal of complementary food, a breastfed infant at 6–8 months needs nine times as much iron and four times as much zinc as a male adult (Dewey 2013). The greatest challenge for meeting micronutrient needs of breastfed children typically occurs during the second 6 months of life. Infants should receive the most nutrient‐rich foods available in the household, yet often the opposite is the case in low‐income settings where they are typically fed nutrient‐poor, watery porridges. Gaps in nutrient intake at this age are generally greatest for iron and zinc (Vitta & Dewey 2013), but other nutrients may be problematic (such as calcium, vitamin A and certain B vitamins) depending on the types of foods consumed (Dewey 2013). Even if breastfed infants are given family foods that are nutritionally adequate for the rest of the household, their intake of certain key nutrients is likely to be lower than recommended (Vossenaar & Solomons 2012).

Infants are often fed cereal‐based porridges as the primary complementary food, but heavy reliance on such foods is problematic for several reasons. First, the porridges prepared from cereal flours tend to be very low in energy density, so the child has to consume a large volume in order to meet energy needs. The child's stomach capacity limits how much can be consumed in a single feeding, thus if meal frequency is low, the ‘bulkiness’ of cereal‐based diets can be a limiting factor (Dewey & Brown 2003). Second, if the infant consumes a typical amount of porridge (100–200 kcal day^−1^), the amounts of other nutrient‐rich foods that can be consumed are limited because total energy needs from complementary foods are only 200–300 kcal day^−1^ at 6–12 months. Last, the inhibition of iron and zinc absorption by phytate in cereal‐based diets makes it difficult to meet requirements for those nutrients, which are high during infancy.

The challenges described earlier have affected human populations for thousands of years, ever since the agricultural revolution when farming became the principal means by which food was procured, rather than hunting and gathering wild foods. Estimates based on presumed food intake of hunter‐gatherers suggest that diets before the agricultural revolution ~10 000 years ago were much higher in many nutrients than modern diets (Eaton & Eaton 2000). Pre‐agricultural humans consumed a wide variety of animal‐source foods and wild plant foods, while cereal grains and legumes were minor parts of the diet. Estimates of the nutrient intakes of infants and young children in pre‐agricultural societies, based on breast milk and premasticated foods provided by their mothers, suggest that the typical pre‐agricultural diet was probably more than adequate to meet nutrient requirements (Dewey 2013). The agricultural revolution resulted in a dramatic shift in human diets towards consumption of cereal grains and other starchy foods, which was accompanied by a deterioration in nutritional status in many populations and a reduction in average adult height. In high‐income countries, average height has increased over the past ~100 years in parallel with improved nutrition (e.g. increased consumption of animal‐source foods) and reduced infectious disease. In low‐income populations, however, stunting in height remains widespread.

In South Asia, children's diets are very poor. For example, in Afghanistan, Bangladesh, India, Nepal and Pakistan, the proportion of children 6–24 months old who are fed even a minimally adequate diet ranges from 12% in Afghanistan to 24% in Nepal (Afghanistan Ministry of Public Health & UNICEF 2013; NIPORT *et al*. 2015; Ministry of Women and Child Development (MWCD) 2015; Ministry of Health and Population (MOHP), New ERA, and ICF International 2012; NIPS & ICF International 2013). Low dietary diversity is a major reason for this situation. The latest surveys in these countries indicate that less than 30% of children in this age group were fed foods from at least four of the seven key food groups the day prior to the survey. Correspondingly, the prevalence of stunting among children under 5 years in this region is very high, 38% (UNICEF 2015).

## Strategies to meet nutrient needs during the first 1000 days

The most common options for meeting nutrient needs of pregnant and lactating women in lower‐income populations include dietary diversification and selection of nutrient‐rich foods, fortification or biofortification of staple foods, supplementation with multiple micronutrients and use of fortified food products specifically designed for this target group. Dietary diversification and increased consumption of nutrient‐rich foods is the primary long‐term goal, but this requires resolving barriers posed by limited access and high cost of such foods. Even with a relatively diverse diet, it may still be difficult to meet iron needs in pregnancy. Thus, other strategies have been implemented to help close the nutrient gaps for pregnant and lactating women. One option is fortification or biofortification of staple foods, which can increase intake of certain key nutrients such as iron, zinc and vitamin A. Another option is multiple micronutrient supplementation, which has been extensively evaluated for pregnant women. A third option is promotion of fortified food products that are designed for pregnant and lactating women and contain both micronutrients and macronutrients, thus providing essential fatty acids and high‐quality protein in addition to vitamins and minerals.

There are also several options for meeting nutrient needs of breastfed infants and children during the period of complementary feeding (6–24 months). Again, dietary diversification and selection of nutrient‐rich complementary foods is the main long‐term goal, but access and cost are often barriers, and it is challenging to meet iron needs even with a relatively diverse diet (Vitta & Dewey 2012). For infants and young children, fortification of staple foods that are consumed by the general population (e.g. wheat flour, maize and rice) is unlikely to close a significant portion of the ‘gap’ in micronutrient intake from complementary foods (particularly for iron and zinc), because children under 2 years typically eat very small quantities. For this reason, specialized products targeted at infants and young children have been developed.

Fortified products for complementary feeding have been categorized into three general types (Dewey & Vitta 2013): (1) fortified blended foods; (2) micronutrient powders; and (3) complementary food supplements. Fortified blended foods are typically made from cereals, legumes and sugar or oil and fortified with certain micronutrients. When pre‐cooked, they are convenient because they require little preparation time. In addition, the use of central processing facilitates quality control. The main disadvantages of fortified blended foods are that (1) it is difficult to ensure adequate micronutrient intake from such products because of the large variability in the amount of product consumed (Dewey 2003), (2) the daily ration of such products usually provides a relatively large amount of energy (e.g. 200 kcal day^−1^), which may displace breast milk, and (3) over‐reliance on a single food may reduce dietary diversity and limit intake of animal‐source foods, fruits and vegetables. In part to overcome these disadvantages, micronutrient powders and complementary food supplements have been developed. Micronutrient powders usually contain only vitamins and minerals and are used for home fortification of traditional infant foods. Adding micronutrient powders to complementary foods just before a feeding ensures that the child receives a full daily dose of micronutrients with no nutrient losses due to cooking. Because these products usually contain little or no energy, they will not displace breast milk or other foods, and they are generally less expensive than food‐based options. The disadvantages of micronutrient powders are that it is difficult to include all of the essential nutrients without splitting the sachet into two portions per day, and they generally do not increase energy, fat or fatty acid, or protein content of the diet. In addition, strong communication strategies may be needed to ensure long‐term adherence and compliance with instructions (e.g. mixing the powder with the first portion of food offered so the full dose is consumed). Complementary food supplements are fortified food‐based products to be added to other foods (i.e. for home fortification) or eaten alone to improve both macronutrient and micronutrient intake, such as small‐quantity (e.g. 20 g day^−1^) lipid‐based nutrient supplements (LNS) and fortified full‐fat soy flour (e.g. Ying Yang Bao (Sun *et al*. 2011)). These products can provide essential fatty acids, micronutrients, macrominerals and high‐quality protein (usually from powdered milk), depending on the formulation. As with micronutrient powders, complementary food supplements provide the full intended dose of key nutrients regardless of the amount of staple food typically consumed, and there is no loss in nutrient content due to cooking. Because the energy content is relatively low, these products are unlikely to displace breast milk. However, complementary food supplements are usually more expensive than micronutrient powders, so it is important to evaluate whether the potential benefits of increased energy density, essential fatty acid content and protein content of complementary foods are important in the target population.

## Impact of prenatal nutrition interventions

In low‐income and middle‐income countries, ~20% of stunting in children under 5 years of age is attributable to small size at birth (Christian *et al*. 2013), although this percentage will likely vary considerably across populations. Thus, it is critical to understand the potential impact of prenatal nutrition interventions on fetal linear growth and newborn stunting. Unfortunately, many studies have reported only birth weight, not length, so the evidence to directly evaluate this question is sparse.

Girard & Olude recently evaluated the impact of nutrition education and counselling during pregnancy on birth weight and other pregnancy outcomes (Girard & Olude 2012). In a meta‐analysis of 13 studies, there was an increase in mean birth weight (+105 g), but this was significant only when nutrition education/counselling was coupled with nutrition support in the form of food supplements, micronutrient supplements or nutrition safety net interventions. The authors did not report on birth length.

Numerous trials have examined the effect on birth outcomes of prenatal multiple micronutrient (MMN) supplementation, and results have been summarized in two meta‐analyses (Fall *et al*. 2009; Ramakrishnan *et al*. 2012). In the first meta‐analysis (Fall *et al*. 2009), there was a small but significant effect on mean birth weight (+22 g) and an 11–17% reduction in the incidence of low birth weight (<2500 g) but no significant effect on birth length (+0.06 cm). In the second meta‐analysis (Ramakrishnan *et al*. 2012), there was a significant (+53 g) effect on mean birth weight and a 14% reduction in low birth weight; data on birth length were not presented. Results from a recent large trial of MMN supplementation in rural Bangladesh (*n* = 28 516 live‐born infants; West *et al*. 2014) were very similar to the estimates from the 2012 meta‐analysis: a significant (+54 g) effect on mean birth weight, a 12% reduction in low birth weight and a small (+0.2 cm) but significant effect on birth length. In all of these trials, the effect of MMN supplementation may have been underestimated because the control group usually received iron and folic acid tablets, which may also have had an impact on fetal growth (Imdad & Bhutta 2012a).

Balanced energy–protein supplementation of pregnant women is another intervention strategy that has been evaluated in several populations. In a meta‐analysis published in 2003 (Kramer & Kakuma 2003), there was a significant effect on mean birth weight (+38 g) but not birth length (+0.1 cm). In an updated meta‐analysis in 2012 (Imdad & Bhutta 2012b), the increase in mean birth weight was somewhat larger (+73 g) and there was a 32% reduction in low birth weight; data on birth length were not reported.

There have been only a few evaluations of fortified food products specifically designed for pregnant and/or lactating women. Three studies to date have reported the impact of LNS designed for this target group. One was a trial comparing medium‐quantity LNS (373 kcal day^−1^) with MMN tablets, both given prenatally (*n* = 1296) (Huybregts *et al*. 2009). While the difference in birth weight between groups was not significant (+31 g, *P* = 0.2), birth length was significantly greater in the LNS group (+0.46 cm, *P* = 0.001). The same research group previously showed that MMN (vs. the control) increased birth length by 0.36 cm (Roberfroid *et al*. 2008); thus, the total predicted impact of LNS vs. control would be 0.46 + 0.36 = 0.82 cm. The effect on birth length of LNS vs. MMN was greater in higher‐risk mothers [those with body mass index (BMI) < 18.5 kg m^−2^ and those with anaemia at baseline]. Two studies have examined the effect on birth outcomes of small‐quantity LNS (118 kcal day^−1^) compared with MMN tablets or iron and folic acid (Adu‐Afarwuah *et al*. 2015; Ashorn *et al*. 2015). In Ghana (Adu‐Afarwuah *et al*. 2015), there was a significant (+85 g) difference in birth weight compared with the iron and folic acid group, but the difference in birth length was not significant in the study group as a whole. However, among first‐time mothers (who had lower BMI and were more likely to be anaemic and have malaria at baseline than multiparous women), infants in the LNS group had greater birth length (+0.91 cm), head circumference (+0.5 cm) and birth weight (+237 g) compared with the iron and folic acid group; similar differences were found when comparing the LNS and MMN groups among first‐time mothers. In Malawi, there were no significant intervention group differences in birth size except for arm circumference (which was larger in the LNS group), but among women with educational levels below the median (<4 years), the proportion of infants with newborn stunting was significantly lower in the MMN (10.3%) and LNS (14.9%) groups than in the iron and folic acid group (22.5%).

In a recent cluster‐randomized effectiveness trial (the Rang‐Din Nutrition Study, clinicaltrials.gov NCT01715038) conducted within a community health program in rural Bangladesh, 4011 pregnant women were randomly assigned to receive small‐quantity LNS or iron and folic acid during pregnancy (Mridha *et al*. 2016). There was a significant effect of LNS on mean birth weight (+41 g) and birth length (+0.2 cm) and a reduction in the percentage of infants who were stunted at birth (18.6% vs. 22.6%) and who had a head circumference z‐score <−2 at birth (20.5% vs. 24.9%). The effects of LNS on newborn stunting were greatest in infants born before a 10‐week interruption in LNS distribution (16.1% vs. 23.0%) and in infants born to women ≤24 years of age or with household food insecurity.

All four of the aforementioned trials using LNS have demonstrated heterogeneity in response, with greater impact seen in more vulnerable women. This is important with regard to the potential for targeting of prenatal nutrition interventions to women who are most likely to benefit.

There is considerable interest in whether nutrition interventions that begin *before* conception have a larger impact than interventions that begin during pregnancy, given that many women in lower‐income countries do not seek antenatal care until the second trimester or later. Although several such trials are underway, few published data are available. Results from the Mumbai Maternal Nutrition Project were recently published (Potdar *et al*. 2014). In this randomly controlled efficacy trial, all women were scheduled to receive a daily snack beginning at least 3 months prior to pregnancy and continuing until delivery; the intervention group received snacks (164 kcal day^−1^) that included green leafy vegetables, fruit and milk and contributed 10–23% of the recommended intakes for six micronutrients, whereas the control group received snacks made from low‐micronutrient foods (potato and onion; 88 kcal day^−1^). In total, 6513 women were enrolled, of whom 2291 became pregnant; newborn measurements were available for 1360 infants. There was no overall effect on birth weight (+26 g, *P* = 0.22) or length (data not shown) in the sample as a whole, but among those who actually started supplementation at least 3 months before pregnancy, there was a significant effect on birth weight (+48 g) and percentage with low birth weight (34% vs. 41%). Contrary to expectations, these effects were seen only in women with higher BMI at enrollment (≥18.6 kg m^−2^) and not in underweight women. Additional evidence on interventions that begin pre‐conception is needed before any conclusions can be drawn regarding programmatic implications.

## Impact of post‐natal nutrition interventions

There are two key periods during the post‐natal ‘window of opportunity’: 0–5.9 months, when exclusive breastfeeding is recommended, and 6–23.9 months, when interventions to improve complementary feeding are usually implemented. Although exclusive breastfeeding during the first 6 months has a significant impact on infant morbidity and survival, there is little evidence to date of an impact on stunting when considering evidence from randomized trials to promote exclusive breastfeeding (Bhutta *et al*. 2008; Black *et al*. 2013). However, a significant effect on linear growth may be difficult to detect unless the study population has a high rate of infection in early life, when exclusive breastfeeding may promote growth by reducing infection. Some observational studies support this linkage (e.g. Engebretsen *et al*. 2008), but it is difficult to rule out reverse causation (i.e. that sicker infants are more likely to be supplemented with non‐breast milk fluids or foods). Because there is insufficient evidence to evaluate the impact of exclusive breastfeeding during the first 6 months on stunting, the remainder of this section will focus on complementary feeding interventions.

Overall, complementary feeding interventions have strong potential for a major impact on stunting, but the evidence to date is mixed (Bhutta *et al*. 2013; Dewey & Adu‐Afarwuah 2008).

Educational interventions to improve complementary feeding practices are often effective at changing behaviours, but most have shown either no impact or only a modest effect on linear growth (Dewey & Adu‐Afarwuah 2008). An exception was a cluster‐randomized trial conducted in Peru (Penny *et al*. 2005) in which stunting at 18 months of age was 5% in the intervention group, compared with 15% in the control group. That trial emphasized three key messages, one of which was consumption of nutrient‐rich animal‐source foods, and the study was conducted in a population where animal‐source foods were available and affordable. Two other studies, in China (Shi *et al*. 2010) and in India (Vazir *et al*. 2013), have also demonstrated the potential to reduce stunting via educational approaches emphasizing dietary diversity and consumption of animal‐source foods, although in both cases, the impact on linear growth was quite small.

The cluster‐randomized trial in India (Vazir *et al*. 2013), conducted in Andhra Pradesh, is worth highlighting because it included education on both nutrition and child development. There were three intervention groups (*n* = 200 mother–infant pairs per group): (1) control [routine Integrated Child Development Services (ICDS)]; (2) ICDS plus 11 messages on breastfeeding and complementary feeding (CF group); and (3) ICDS plus 11 messages on breastfeeding and complementary feeding and additional education and play groups for responsive feeding and psychosocial stimulation (RCF&PG group, 27 messages in total). Both intervention groups received bi‐weekly visits by trained village women for 12 months, when infants were between 3 and 15 months of age. Infants in the CF group (but not the RCF&PG group) had greater length gain than infants in the control group, but the differences in stunting at 15 months were not significant (37% vs. 28% vs. 36% in control, CF and RCF&PG, respectively). The mental development score at 15 months was higher in RCF&PG children than in control children (+3.1 points). The authors observed that micronutrient intakes were low in all groups, despite increases in energy and protein from complementary foods in the intervention groups. This situation, plus the possibility that the number of messages in the RCF&PG may have been overwhelming, may account for the lack of growth response in the RCF&PG group.

In some populations, low energy density of complementary foods may limit energy intake. Interventions to increase the energy density of complementary foods have yielded mixed results. Of five studies previously reviewed (Dewey & Adu‐Afarwuah 2008), two had a positive impact on linear growth but three had no impact on energy intake or growth. Increased energy density may be effective when the traditional complementary food has a low energy density and infants are unable to compensate by increasing the volume of food consumed or feeding frequency, but otherwise, this strategy is not likely to affect growth.

Fortification of complementary foods with micronutrients via central processing or home fortification strategies (such as micronutrient powders), without any additional macronutrients (energy, protein or fat), has generally not affected linear growth (Dewey & Adu‐Afarwuah 2008, Ouédraogo *et al*. 2010, CIGNIS Study Team 2010, De‐Regil *et al*. 2011). Similarly, strategies to increase bioavailability of key nutrients such as iron and zinc have generally failed to reduce stunting (Mamiro *et al*. 2004; Mazariegos *et al*. 2010). One exception is a randomized trial conducted in India (Dhingra *et al*. 2004) where milk powder (fortified or unfortified) was given for 1 year to 465 children whose average age was 23 months. The group given the fortified milk had significantly less morbidity and greater weight and height gain than the group given unfortified milk. In this trial, the use of milk powder as the food vehicle for the extra nutrients may have increased the likelihood of a positive growth response to a fortified product, because milk supplies substantial amounts of growth‐promoting macronutrients and does not contain ‘anti‐nutrients’ such as phytic acid (which can interfere with absorption of critical nutrients).

Numerous interventions have included the provision of complementary foods or a food product offering extra energy (with or without added micronutrients), alone or in combination with some other strategy such as education for caregivers. Some, but not all, of these interventions have had a positive impact on linear growth (Dewey & Adu‐Afarwuah 2008). There has been a wide range of impact, from trivial to relatively large, which may reflect variations in the target populations' food security and the nutrient quality of the food provided. Three studies have directly compared provision of food coupled with an educational intervention vs. education only (Bhandari *et al*. 2001; Roy *et al*. 2005; Christian *et al*. 2015). All were conducted in South Asia, and in all cases, there was a greater impact when the package included food.

## The need for integrated approaches

It is becoming increasingly evident that nutrition interventions, by themselves, may not result in the desired impact if the target population suffers from frequent infection, both clinical and subclinical (i.e. asymptomatic). It is well known that infections can cause linear growth retardation, but subclinical conditions such as environmental enteric dysfunction, inflammation and other physiological responses to environmental insults (including mycotoxins and household air pollution) are likely to be far more common than clinically obvious infections and may account for a large proportion of stunting (Dewey & Mayers 2011; Khlangwiset *et al*. 2011; Smith *et al*. 2011; Prendergast & Kelly 2012; Smith *et al*. 2012). Research studies are uncovering the mechanisms by which these conditions adversely affect linear growth – both *in utero* and post‐natally. Meanwhile, the reduction of stunting requires strategies to prevent and manage infection and to reduce exposure to the putative causative agents of subclinical conditions that impair growth. It is likely that the combination of these strategies with appropriate nutrition interventions will be most effective, as adequate nutrition can reduce the negative impact of infections on growth by strengthening the immune system, providing extra amounts of nutrients to compensate for effects of infection and allow for catch‐up growth, preventing poor appetite caused by nutrient deficiencies, and favouring the growth of beneficial bacteria in the gut that enhance gut function and immune defences (Dewey & Mayers 2011).

A few large‐scale trials are underway that combine nutrition and infection control. These include the WASH Benefits Trial (water, sanitation and hygiene interventions: singly, combined or in combination with nutrition interventions – http://www.washbenefits.net/) in Bangladesh and Kenya and the Sanitation, Hygiene, Infant Nutrition Efficacy Project in Zimbabwe (independent and combined effects of improved water, sanitation and hygiene and improved infant feeding http://www.sdc.admin.ch/en/Home/Projects/Project_Detail?projectdbID=218331). Both trials target mainly the post‐natal period. Encouraging results were recently published from a cluster‐randomized trial in Burkina Faso in which the intervention group received a ‘package’ that included provision of small‐quantity LNS to infants between 9 and 18 months of age together with weekly home visits that included morbidity surveillance and treatment of diarrhoea and malaria (Hess *et al*. 2015). There was a 25% reduction in prevalence of stunting at 18 months (29% vs. 39% in intervention vs. non‐intervention children) and significant positive effects on motor, language and personal–social development.

Enhancing child development, not just physical growth, should be an integral part of strategies to reduce stunting and its longer‐term sequelae with regard to human capital (Hoddinott *et al*. 2013). Integrated interventions that include components to improve early child development via psychosocial stimulation, responsive feeding and care for mothers and children may result in a larger impact on growth and behavioural development than would be expected from single interventions (WHO 1999; Yousafzai *et al*. 2013). Thus, the ideal package would combine improved nutrition, infection control (including WASH) and interventions to enhance child development and would tackle the entire ‘window of opportunity’, i.e. both the prenatal and post‐natal periods. To date, this type of comprehensive approach has not yet been attempted in efficacy or effectiveness trials.

## Summary and conclusions

In regions such as South Asia, meeting nutrient needs during the first 1000 days is a major challenge. Pregnant and lactating women and their young children need diets with high micronutrient density, but in low‐income populations, intakes are usually well below recommended amounts for several key nutrients because diets are dominated by staple foods with low nutrient density and poor mineral bioavailability. Gaps in nutritional adequacy in such populations probably date back to the agricultural revolution ~10 000 years ago. Prior to that time, stunting was less common and intakes of key nutrients were likely considerably higher than observed today. For modern populations relying on predominantly cereal‐based diets, nutrient deficiencies and stunting are widespread.

Several options for improving diets of pregnant and lactating women and their infants exist, including dietary diversification and increased intake of nutrient‐rich foods, improved complementary feeding practices, micronutrient supplements and fortified foods or products specifically designed for these target groups. Evidence from intervention trials indicates that several of these strategies, both prenatal and post‐natal, can have a positive impact on child growth. However, there is considerable heterogeneity in growth response to such interventions, which is likely to be related to the potential to *benefit* (i.e. is the population malnourished?) as well as the potential to *respond* (i.e. are there other factors constraining the ability to increase linear growth?) to improved nutrition.

Thus, to design effective strategies to reduce stunting, we need to better understand the mechanisms underlying both prenatal and post‐natal growth restrictions. In particular, research is needed to examine the consequences of clinical and subclinical infection and inflammation, the role of the microbiome, the impact of environmental contaminants (e.g. aflatoxin and household air pollution), the importance of specific nutrients required for lean body mass deposition and other food constituents, the influence of maternal mental health and caregiver behaviours, and the long‐term effects of prenatal nutrition and epigenetic influences on growth and development of offspring.

Meanwhile, there are several policy and programmatic implications of the evidence available to date. First, because a considerable proportion of stunting occurs before birth, nutrition interventions should cover both pregnancy and the post‐natal period. Second, nutrition interventions are likely to have a greater impact on growth if they are delivered as part of a package of interventions that address the multiple causes of stunting. This means that attention should be paid to prevention and control of prenatal and post‐natal infection and subclinical conditions that restrict growth, care for women and children, and stimulation of early child development. This is particularly relevant for South Asia, where it is common for women to be poorly nourished both prior to and during pregnancy, poor hygiene and sanitation are widespread problems, and the prevalence of child stunting is very high. These conditions, combined with the large population of the region, mean that efforts to reduce stunting globally must include an emphasis on South Asia. Integrated interventions that simultaneously address all of these factors hold great promise for reducing stunting and enhancing human capital formation in this region and elsewhere.

## Source of funding

There were no external funding sources for this research.

## Conflicts of interest

The author declares that she has no conflicts of interest.
